# Indigenous mothers’ experiences of using primary care in Hamilton, Ontario, for their infants

**DOI:** 10.1080/17482631.2019.1600940

**Published:** 2019-04-29

**Authors:** Amy L. Wright, Susan M. Jack, Marilyn Ballantyne, Chelsea Gabel, Rachel Bomberry, Olive Wahoush

**Affiliations:** a Lawrence S. Bloomberg Faculty of Nursing, University of Toronto, Toronto, Canada; b School of Nursing, McMaster University, Hamilton, Canada; c Holland Bloorview Kids Rehabilitation Hospital, Toronto, Canada; d Department of Health Aging and Society, McMaster University, Hamilton, Canada

**Keywords:** Primary health care, health services, Canada, qualitative research, health equity, maternal/child health, Indigenous mothers and infants

## Abstract

**Purpose**: Access to primary care can help mitigate the negative impacts of social inequity that disproportionately affect Indigenous people in Canada. Despite this, however, Indigenous people cite difficulties accessing care. This study seeks to understand how Indigenous mothers—typically responsible for the health of their infants—living in urban areas, experience selecting and using health services to meet the health needs of their infants. Results provide strategies to improve access to care, which may lead to improved health outcomes for Indigenous infants and their families.

**Methods**: This qualitative interpretive description study is guided by the Two-Eyed Seeing framework. Interviews were conducted with 19 Indigenous mothers and 5 primary care providers.

**Results**: The experiences of Indigenous mothers using primary care for their infants resulted in eight themes. Themes were organized according to three domains of primary care: structural, organizational and personnel.

**Conclusions**: Primary care providers can develop contextual-awareness to better recognize and respond to the health and well-being of Indigenous families. Applying culturally safe, trauma and violence-informed and family-centred approaches to care can promote equitable access and positive health care interactions which may lead to improved health outcomes for Indigenous infants and their families.

The use of health services is an important way to promote health and well-being. Despite this, however, many Indigenous people in Canada experience inequitable access to health care, citing barriers such as racism, a fear of judgement and a lack of traditional Indigenous health services (The Truth and Reconciliation Commission of Canada, 2015). Primary care is one of several types of health services available in Canada, and refers to the community-based comprehensive provision of health promotion and management of non-emergency and chronic health conditions across the lifespan (Canadian Nurses Association, 2015). While the use of primary care is important at every life stage, primary care has important implications for infants, as it is associated with reduced infant mortality (Brandon, Costanian, El Sayed, & Tamim, 2016). Indigenous infants in Canada experience poorer health outcomes and higher rates of infant mortality than non-Indigenous infants, and yet how they access and use health services remains largely unknown (Smylie, Fell, Ohlsson, & Joint Working Group on First Nations, Indian, Inuit and Métis Infant Mortality of the Canadian Perinatal Surveillance System, 2010; Wright, Wahoush, Ballantyne, Gabel, & Jack, 2018).

This article reports part of a broader study that explored how Indigenous mothers living off-reserve in a city experience the phenomenon of selecting and using health care services to meet the health needs of their infants and included 31 participants (Wright, 2019). Participants included 19 mothers, five primary care providers (PCPs) and seven providers of early childhood development services.^1^ The results presented in this article reflect the data as they relate specifically to the experiences of selecting and using primary care. The objective of this research is to help inform primary care delivery and health policy, which may lead to more equitable access to primary care for Indigenous mothers and infants.

## Background

The social determinants of health such as income, education, gender and access to health care, play a key role in the health of individuals (Reading & Wien, 2009). In addition, racist and discriminatory policies stemming from the impact of colonization mean that Indigenous people in Canada are particularly impacted by poor health outcomes resulting from social inequities (Browne et al., 2012; The Truth and Reconciliation Commission of Canada, 2015). Adequate access to primary care is one important way to mediate the effects of inequities that result in higher rates of disease, injury and mortality for some Indigenous infants (Reading & Wien, 2009; Reading & Halseth, 2013; Smylie et al., 2010). Yet despite these potential benefits, 13% of mothers in Canada report difficultly accessing care for their infants (Brandon et al., 2016). An earlier study with First Nations parents in Hamilton, Ontario, reported barriers that included long wait lists, transportation problems and an inability to afford health services (Smylie et al., 2011).

Mothers are most commonly responsible for making health-related decisions for their children (Minkovitz et al., 2005), and, thus, understanding their experiences of how they select and use health care for their infants is imperative for providing care that better meets the health needs of their infants. Research with Indigenous mothers in Canada’s urban areas is particularly important, as a recent census showed that 56% of Indigenous people live in urban areas, and that more than half of these are women (Government of Canada, 2014; Statistics Canada, 2018).

## Methods

This qualitative research study was guided by interpretive description (ID) as developed by Thorne (2016), and the application of Two-Eyed Seeing to ensure the inclusion of both non-Indigenous and Indigenous worldviews (Bartlett, Marshall, et al., 2012). The study was philosophically grounded in constructivism and naturalistic inquiry and underwent review and approval by three ethics bodies: the Hamilton Integrated Research Ethics Board, Mohawk College Research Ethics Board, and the McMaster University Family Medicine program.

Founded in nursing epistemology and disciplinary goals, ID emphasizes development of usable knowledge that offers practical application for clinicians (Thorne, 2016). Two-Eyed Seeing stresses the importance of viewing the world through both Western (mainstream) and Indigenous worldviews (Bartlett, Marshall, et al., 2012). The application of Two-Eyed Seeing to this study has resulted in a community-based approach to research, in which the research question and study development were informed by the Indigenous community, a Métis Scholar who sat on the researcher’s Supervisory Committee, and a First Nations nurse who worked as a research assistant alongside the researcher throughout the study. Examples of the influence of community involvement in the research process included: offering mothers the optional presence of the research assistant during interviews, not offering tobacco (as a sign of respect) as not participants were familiar with or practiced this custom, offering a cash honorarium for participation, collaborative data analysis (as will be discussed below) and community-driven knowledge and dissemination activities, among others. Further details of how Two-Eyed Seeing was applied to the study will be available in a future publication.

### Reflection of the researcher

When undertaking a qualitative study—and congruent with Indigenous approaches to research—it is recommended that the researcher reflect on their beliefs, values and motivations (Lavallée, 2009). Briefly, the first author is a non-Indigenous nurse practitioner of European settler ancestry. She acknowledges that her upbringing has been dominated by mainstream Western culture, and that as such, she benefits from unearned privilege. She seeks to engage in ethical interactions between members of Indigenous and Western cultures in Canada. As a nurse researcher, she seeks to co-create knowledge through understanding the experience of others, valuing similarities and differences, and collaboratively creating wisdom that will benefit all (Bartlett, Marshall, & Marshall, 2012).

### Setting and sampling techniques

This study took place in the city of Hamilton, Ontario, located on the traditional territories of the Haudenosaunee and Mississauga nations (Ontario Federation of Labour & Ontario Federation of Labour Aboriginal Circle, 2017), and close to two reserves including Six Nations of the Grand River and Mississaugas of the New Credit. Indigenous people make up 1.7% of the Hamilton population, and Indigenous children aged 14 and younger represent 2.8% of all children (Statistics Canada, 2015). The majority of First Nations residents report living below the poverty line, and face high rates of diabetes, hepatitis C, hypertension and mental health concerns (Smylie et al., 2011). Although most First Nations children (83%) are said to have been seen by a PCP in the past year, many First Nation parents state concerns about their child’s physical and mental/emotional well-being, and nearly 5% with children younger than five view their children’s health as only fair or poor (Smylie et al., 2011).

A purposeful sample of Indigenous mothers who met the following inclusion criteria were invited to participate: (a) self-identified Indigenous ancestry; (b) parenting an infant less than two years of age; and (c) living in Hamilton, Ontario. Snowball sampling was used by asking participants to provide names of contacts with similar experiences. Within the study, theoretical sampling informed decisions to explore aspects of the phenomenon arising from interactions with participants that would require further depth (Patton, 2015).

The initial goal was to recruit a sample of 30, or to recruit until data redundancy was realized, and the emerging themes demonstrated information power—that is, the ability of the data to provide a comprehensive understanding of the phenomenon under study (Malterud, Siersma, & Guassora, 2015). Recruitment procedures consisted of poster invites, flyers, and word of mouth. To develop a deeper understanding of the contextual factors influencing the phenomenon, we triangulated our data by including experiential knowledge by interviewing PCPs. A purposeful sample of providers who worked in primary and early childhood services with Indigenous mothers and infants in Hamilton were invited to participate. The contextual data provided by PCPs is described in this article, and contextual data from health providers in early childhood development services is described in another publication (Wright, Jack, Ballantyne, Gabel, & Wahoush, in press). Health providers were recruited via email and phone invitations, and by word of mouth.

### Data collection

Data were collected using semi-structured, one-on-one interviews with mothers and PCPs and a final discussion group with mothers. These strategies were used to honour the oral tradition which is important to Indigenous people (Kovach, 2009). The interview guide was developed in partnership with the research assistant to ensure questions were presented in culturally safe ways. All mothers completed a single interview, lasting approximately one hour, at a location of convenience for them. This was most often in their home, or at the Indigenous Friendship Centre (IFC). The interview guide was designed using Andersen’s *Behavioral Model and Access to Medical Care* (1995), which describes the variables associated with access to health care and has been extensively tested and validated. The model suggests that environmental factors, characteristics of the population, health behaviour and health outcomes work together to influence how an individual interacts with and uses, health services (Andersen, 1995).

The interviews with mothers were augmented by ecomaps, which originated from systems theory and ecology, and have been used by social workers and nurses in clinical practice to visually depict family supports and resources (Hartman, 1995; Stewart & Allan, 2013). When used as a method of data collection in research, it is a collaborative way to engage participants in conversation about which services they access or use, and to help them identify the nature or quality of support received from that service (Rempel, Neufeld, & Kushner, 2007; Stewart & Allan, 2013). Sitting side-by-side when making the ecomap was helpful in limiting power inequalities between the researcher and mother. The use of ecomaps helped to keep the interview focused, as they provided a visual reminder of what had and had not been discussed.

Interviews with PCPs took place with the researcher at a location convenient for the PCP, most often in an office or over the phone, and lasted approximately one hour. All PCPs completed a single interview. The questions were developed to elicit additional contextual details about the themes emerging from the mothers’ data. Data from interviews with PCPs did not undergo a separate analysis, but rather, this data were used to complement and triangulate the mothers’ data.

Finally, once all data were collected, and the initial analysis complete, a discussion group was held with the mothers to provide an opportunity for member checking and to further expand on and clarify any concepts that had emerged. Member checking is not required in ID (Thorne, 2016) but was essential to validate the researcher’s interpretations of the mothers’ experiences in the absence of Indigenous lived-experience. All 19 mothers were invited to attend and eight joined. Mothers were asked to confirm themes emerging from the data and were given an opportunity to clarify or remove data they felt was misunderstood. They did not ask for any data to be removed.

All interviews and the group discussions were audio-recorded for transcription and analysis using NVIVO 12 (QSR International, 2018). Field notes provided insight into interactions between the researcher and the participants, the research setting, details relating to context, and the influence of the physical environment on participants (Mulhall, 2003). All data were stored in the researcher’s locked office.

### Data analysis

A thematic data analysis was undertaken, guided by Thorne (2016), Two-Eyed Seeing (Bartlett, Marshall, & Marshall, 2007) and by consulting the ecomaps. Data analysis in ID is not merely a description of the data, but rather an interpretation of the patterns found within them, and the relationships and interactions between the patterns (Thorne, 2016). The final product of an ID strives present the phenomenon in a new way that furthers clinicians’ understanding along with new and meaningful ways to apply the knowledge to their clinical work (Thorne, 2016). Through the application of Two-Eyed Seeing, data analysis was initially completed independently by both the researcher and the research assistant. Both then came together many times to compare results and to clarify each other’s interpretations of the data, so that consensus could be reached on the presence of codes and eventual themes. The research assistant provided invaluable insight into local customs and traditions that were at play. In this way, Two-Eyed Seeing was used to promote the interweaving of Western and Indigenous ways of knowing.

The analysis began during data collection and continued through the final, more formal analytic phases (Thorne, 2016). Only broad-based and generic coding strategies were applied until the researcher and research assistant were fully engrossed in the data and understood the relationships between various elements within the phenomenon. Data from the PCPs were analyzed to provide additional context to emerging themes in the mothers’ data. Finally, the ecomaps were used as a visual confirmation of the analyzed transcripts, including information about which services were used by whom.

Themes were organized using three discrete domains of primary care as described by Hogg, Rowan, Russell, Geneau, and Muldoon (2008): structural, organizational and personnel. The structural domain is composed of system-level elements that influence care delivery, including policies, funding and governance and community-level policies, population characteristics and infrastructure (Hogg et al., 2008). The organizational domain refers to policies and factors that influence the provision of care, while the personnel domain refers to factors relating specifically to PCPs and how they provided care (Hogg et al., 2008).

## Results

The findings in this article represent the experiences of 24 individuals; 19 mothers and five PCPs (family physicians/nurse practitioners). Data from interviews with additional seven providers of early childhood development services will be presented elsewhere (Wright et al., in press). Of the participant mothers, 15 identified as First Nations, two identified as Métis, and two were unsure of their specific Indigenous culture. The median age of the mothers was 28 years and approximately one-third were first-time mothers. All but one infant had a regular PCP. See Table I: Demographic information: Participant mothers. One PCP identified as First Nations, and all other PCPs were non-Indigenous.

**Table I.** Demographic information: participant mothers.

Variable	Category	Frequency (%)
Age	<25 years	5 (26)
	26–30 years	8 (42)
	>31 years	6 (32)
Number of Children	First time moms	5 (26)
	2–5 children	14 (74)
Education	Less than High school	9 (47)
	Completed only high school	3 (16)
	Some College/University	7 (37)
Marital Status	Single/Separated	9 (47)
	Married/Common-law	10 (53)
Indigenous Identity	First Nations	15 (78)
	Métis	2 (11)
	Inuit	0 (0)
	Unknown Indigenous culture	2 (11)
Income	Full-time Employment	7 (37)
	Ontario Works (social assistance)	10 (53)
	Disability Pension	2 (10)
Change of address during life of infant	Moved at least once	10 (53)
	Same residence	9 (47)
Regular Health Care Provider	Family physician	17 (90)
	Pediatrician	1 (5)
	None	1 (5)

N = 19.

Results are presented in three stages below. Descriptions by the participant mothers of how they selected PCPs are articulated, followed by the circumstances in which they used PCPs and finally their experiences of using primary care to meet the health needs of their infants.

### PCPs are selected based on convenience

When asked how they select primary care services to meet the health needs of their infants—including the provision of vaccinations or treating illnesses—mothers described selecting a PCP based on convenience. Mothers were an existing patient of the PCP they chose to care for their infant, or if they did not have a PCP, they sought a new one based on recommendations from a family member or friend. In the absence of these recommendations, mothers chose a new PCP simply because the primary care provider was accepting patients and available. As one mother explained, “I found him in the phonebook. At the time he was a new doctor and he was looking for patients. My sister and a couple of people I knew go to the [clinic] down there, so I figured why not?”. No mothers in this study described investigating a PCP for quality, acceptability, or their ability to meet their infant’s health needs prior to becoming a patient.

### How mothers use primary health services to meet their infant’s health needs

When asked how they use their PCP to meet their infant’s health needs, mothers described using their PCP for: (a) *routine care*; (b) *health education*; and (c) *non-emergent treatment*. All mothers described using their PCPs to access routine infant care, including vaccines, assessment of appropriate growth and development, and well-baby check-ups. The provision of newborn health education by PCPs was inconsistent, with some mothers receiving this, while others did not. Those who did not receive education from their PCP sought information elsewhere (the internet, public health services or other early childhood development services). Experienced mothers wanted their infant’s PCP to initiate health education, regardless of their parenting experience, because they had received this education many years previously. One mother expressed her need for a reminder of basic health information for her newborn: “I haven’t had a baby in four years and I was back to square one. I was nervous…”. In contrast to the reported experiences of these mothers, the PCPs interviewed in this study shared that they routinely provided health education to all mothers.

Mothers used their infant’s PCP for routine health services as well as for meeting any non-emergent health needs. These included a visit to their infant’s PCP for a fever, cold, ear or eye infections, breathing issues and skin disorders. Mothers preferred to see their infant’s PCP for these non-emergent issues rather than seek care at emergency departments (EDs), however they would attend the ED if they were dissatisfied with the PCP’s care, or if they were unable to get an appointment.

### Experiences of using primary health services

Eight themes relating to how mothers experienced using primary care for their infants were organized according to the three domains of primary care. A summary of these themes can be found in Table II: Thematic summary. The following describes each of these themes in more detail, using the voice of participants.

**Table II.** Thematic summary.

Domain	Theme
Structural	Neighbourhood influences health Multi-service clinic
Organizational	Flexible appointments Alternative options for care Welcoming receptionists Welcoming spaces
Personnel	Relationships are key
	Approaches to care

#### Structural

Mothers described two themes within the structural domain, including: (a) *neighbourhood influences health*; and (b) *multi-service clinics*.

##### Neighbourhood influences health

Mothers described how Hamilton’s economically deprived neighbourhoods in which they lived were characterized by high rates of crime and that living there was detrimental to their health. They were unable to comfortably leave their homes with their infants after dusk for fear of violence from members of the community, which limited their ability to exercise and be outdoors. They suggested that living in these neighbourhoods negatively influenced their infant’s PCP such that PCPs demonstrated feelings of apathy towards the community by running “ghetto” and unwelcoming clinics. One mother described her experience of attending a run-down clinic in her neighbourhood:

…you have to expect living in this area you‘re not going to get the best healthcare. It seems like they care less when you‘re in a poverty-stricken area…the doctor’s office is kind of ghetto looking. They just threw it together it kind of seems… It doesn’t feel personable, it doesn’t feel welcoming, it doesn’t feel warm, and it feels like you’re in and out, and they are not doing their job. They don’t ask you how you’re doing, as they would in a different nicer area. Ya, I guess in the area you live in you can expect different treatment.

Conversely, mothers who had attended clinics in more affluent neighbourhoods were overwhelmed by spacious clinics that were well-maintained and visually appealing but made them feel out of place.

…It was so nice! Like this is going to sound ghetto. Like a high-class pediatrician because they were giving out free baby Tylenol. Right? And my pediatrician doesn’t do that for me… a lot of parents when I looked around, you can’t help but notice, but they were older, lighter skin parents…They like were more classier looking type of people…I felt a little uncomfortable when we were waiting in there.

Mothers equated the attention and care put into providing a welcoming and clean clinic environment as a reflection of a PCP’s care for their patients. Thus, the participants perceived that delivering services in the dirty and run-down clinics in their neighbourhood meant that PCPs did not care about them.

##### Multi-service clinic

Several mothers expressed that the availability of numerous services within the same facility would improve their experience. Two mothers had accessed primary care clinics that provided a nutritionist, pediatrician and other health providers within the same building as their PCP. Their perceptions of care at these facilities were that they offered better access to specialty services than relying on referrals from their PCPs to other services in the community. Another mother described the ideal primary health service as one that combined early childhood development services with access to PCPs.

#### Organizational

Mothers described four organizational policies that influenced their experiences of using primary care for their infants, including: (a) *flexible appointments*; (b) *alternative options for care*; (c) *welcoming receptionists*; and (d) *welcoming spaces.*

##### Flexible appointments

Mothers stressed the need for flexibility in scheduling appointments and for being respected when they changed or cancelled appointments. Many mothers had several children and were balancing busy and complex lives that occasionally required appointments to be re-booked. Additionally, many mothers did not want to take public transit with a sick infant and were at risk for cancelling appointments if their ride became unavailable. Several clinics penalized those who cancelled appointments by charging fees or threatening to de-roster patients. For mothers already struggling financially, these consequences were devastating. One mother described her experience, including when an office staff threatened to file a report to child protection services if she continued to cancel:

They were kind of like very… pushy about appointments? Like I was also very small when I had her [my daughter], and I was losing weight for some reason and very sick. So they wanted me in every two weeks. But the one appointment they scheduled me for was December 27th. I had Christmas dinner that day, so I called to reschedule it…They are like, “You can’t miss this appointment. If it happens again we are going to have to call [child protection services]” … they were just really rude about it. It kind of sucked. That was like one of the starters in me switching appointments or like switching doctors.

Primary care providers were aware that some patients had competing priorities that influenced their ability to attend appointments and endeavoured to provide flexibility. Often these families were known to the clinic and as a result, they capitalized on any visit the mothers could make, whether by appointment or as a walk-in, getting as much done as possible, such as blood work and physical examinations, amongst others. One family physician working at a primary clinic for women living in the downtown core shared:

I think all of the clinicians understand that those populations really need to, when they call in or they show up, you see them. Because that is a moment that they’re coming and asking for assistance. They probably had a moment to come and see you then. If you don’t capitalize on that you might not be able to see them for a month or so.

Mothers also wished PCPs could see their infant on the same day they called seeking an appointment. They expressed worry about their inability to detect their infant’s subtle signs of illness, so they needed a professional’s opinion. Mothers who were worried about the health of their infants said they would not wait for a later appointment but would rather go to an alternative walk-in clinic or ED to have their child seen right away.

Health providers understood that an infant’s well-being was a source of anxiety for mothers and that they often wanted access to service the same day they called. They did their best to see infants in that situation, but organizational policies and scheduling restrictions made this difficult. In some cases there were no options other than to send mothers and infants to after-hours alternatives.

##### Alternative options for care

When their PCPs were unavailable, families were sent to seek help elsewhere. Many mothers sought care at their neighbourhood walk-in clinics or the ED. Mothers reported that some clinics provided care after-hours into the evening, while others provided on-call triaging services where a physician or nurse practitioner could speak with a mother to determine if care was needed urgently. Some mothers were penalized for seeking care at walk-in clinics; they were charged fines or were threatened with the removal of their infant from the PCP’s roster. A mother shared her dilemma of using a walk-in clinic:

My doctor’s office, they tell you specifically do not go to walk-in clinics because you will be billed for it because they have their own walk-in clinic. Which a lot of patients aren’t happy with because it is only from five-thirty to seven at night, and you’re lucky if you get to see someone because it is so packed.

Despite these consequences, mothers sought care when required. This most often meant going to an ED where they would not be financially penalized by their PCP.

##### Welcoming receptionists

Receptionists were often the point of first-contact and their interaction with families set the tone and perception of the clinic. Positive interactions occurred when the receptionists were welcoming, familiar and friendly, and took care to remember infant’s names. Negative experiences included instances when receptionists were rude or unfriendly, threatened to involve child protection services for cancelling or rescheduling appointments, or when they breached confidentiality. The threat of a report to child protection services was particularly damaging for mothers, who felt shamed, judged and angry after these encounters. Mothers who experienced these threats were uncomfortable returning for care; they felt their parenting was under surveillance and their ability to maintain custody of their children was at risk. Mothers who overheard receptionists speaking with colleagues about a patient’s personal health information felt they too were at risk for having their information shared inappropriately. One mother shared an example of her experience overhearing the inappropriate remarks of a receptionist:

…a patient had called for a refill of painkillers and she [receptionist] got off the phone and started talking to the other receptionist in front of everybody [about] what the girl just called about. And she is like, “Oh yes, she called this many times for [percocet], and I told her she is not getting them.”…they said her name, the girl’s name, and got off the phone… the whole waiting room could hear what they were talking about. So, I wouldn’t appreciate that if somebody was talking like that about my business.

Positive interactions with receptionists were associated with good experiences of using the clinic, while the opposite was true of negative encounters. In some cases, negative interactions resulted in mothers not returning for care; they found another PCP or used alternatives like a walk-in clinic.

##### Welcoming spaces

Mothers expressed the need for welcoming spaces for individuals seeking care, including comfortable waiting rooms, reasonable wait times and an accepting atmosphere for individuals of different cultural backgrounds. Small waiting areas felt crowded to mothers, who found it difficult to keep young children seated while they waited to be seen. Mothers felt small and dirty waiting rooms caused further harm to their infants, as they increased their risks of contracting illnesses from other patients. Long wait times were problematic, as infants who felt unwell were often fussing or crying, bothering others and making mothers feel self-conscious. Mothers suggested PCPs could mitigate these challenges by providing age-appropriate activities in waiting rooms, such as TVs, books, or toys. One mother explained:

Yes, and my doctor is a very thorough man. He likes to explain everything, which is great, and I can see why the backlog happens, because he is like that. But when you have a 6-month old that’s coming in for his first needles and you have to wait an hour and half, and I have held off breastfeeding because I wanted to give it to him once the needles come and he is upset. That is super frustrating.

Mothers also expected clinic to be welcoming to people of all cultures. Some of the mothers felt judged by others for their Indigenous culture and wanted their children to feel welcomed by PCPs. One mother shared:

I feel like if everyone was welcomed no matter what kind of race you were, or color, where you came from I feel like that would be ideal. I feel like it shouldn’t be secluded for just one race or culture. I feel like that is where we go wrong sometimes.

Some suggested creating a dedicated clinic space for displaying art work and languages of different cultures; however not representing Indigenous cultures while incorporating many other cultures was interpreted as racist, and left mothers feeling devalued by PCPs.

#### Personnel

Many characteristics of the PCP enabled or inhibited a positive experience or access to care. These characteristics are grouped in two themes: (a) *Relationships are key*; and (b) *approaches to care*.

##### Relationships are key

Mothers repeatedly emphasized the importance of having a relationship with their infant’s PCP. The building blocks of developing these relationships were woven throughout mothers’ stories, as relationships were important not only for them, but equally important for their children. Relationships were facilitated through building trust, a consistent provider, effective communication skills, and a female PCP. Discriminatory or racist care was detrimental to building relationships.

First, mothers needed to trust their infant’s PCP to feel they were receiving appropriate care and to experience a reciprocal and non-threatening relationship. Misdiagnoses, a lack of professionalism and breaches in confidentiality weakened or damaged the sense of trust mothers had for their infant’s PCP. This then led to negative experiences and to seeking care in other places like the ED. Health providers also recognized the importance of building a trusting relationship with patients. They acknowledged how trust created a comfortable environment allowing mothers to feel open to share their health concerns. One family physician described the importance of trust in her interactions with mothers and infants:

…when they sort of trust what you‘re saying and even if they’re uncomfortable, they know they will be going home with a plan of action. And it is like okay, I am nervous because my child is sick or I am nervous about this or whatever, but I have a good enough relationship with my doctor to know they’re not concerned so I should not be concerned.

Second, mothers described that seeing the same PCP for their infant at each appointment assisted in building relationships and establishing trust. Mothers whose infants saw different care providers at each visit reported that PCPs were often not up to date on their infant’s condition or clinical course. Gaps in information or understanding meant mothers had to explain their infant’s health history at every visit, causing frustration and wasted time.

Third, a PCP’s ability to effectively communicate with mothers and their infants was another important component of building relationships and promoting positive interactions. Effective communication skills included taking the time to listen and acknowledge concerns, valuing mothers as experts of their own children and validating their insight. This made mothers feel they were heard and that their concerns were taken seriously. Mothers who felt they were not listened to were less likely to trust their PCP’s opinions and suggestions and were more likely to seek care elsewhere.

Fourth, seven mothers shared that they felt more comfortable with a female PCP, and spoke of their inherent trust of women, who they perceived as generally more emotional and caring. They related this to their own histories of violence with men, and their desire for their children to grow to respect women. One mother described how a child who grows up surrounded by women is given the best chance to learn to respect them, and to be cared for and nurtured emotionally.

I feel like a baby his age, especially when there has been so much violence against women. I have been a victim of it…I feel like if you are in an environment full of women you learn to respect women. I don’t want him to grow up and be disrespectful. I want him to grow up and appreciate the women around him…I feel like if we had access to a doctor’s office…with all female staff that would be ideal in my opinion.

Finally, some mothers described traumatic experiences of unethical care related to racism and discrimination, which damaged the potential for building trusting relationships. Mothers who experienced racism and discrimination attributed this to their PCP’s assumption that they were Indigenous—based on their appearance and/or last name. One mother described how she believed her children’s appearance and last name led to their higher risk of experiencing racism:

When you look at my babies you know they are Native American. When you look at my older ones, oh my goodness they got freckles. They are cute and they just look like little Caucasian babies right? But my younger ones, they don’t…it‘s very hard for me to wrap my head around how somebody could treat me the way they did because of who, not even who I am…but just because of my race. I think I am crying too because I am scared. I am scared for them. There isn’t a day that doesn’t go by when I think about things that they have to go through just because of who they are.

Other mothers felt discriminated against for having children at a young age. They perceived that their PCP believed they were unfit to parent because they were young. One mother described how her age prevented her from giving informed consent:

…They wouldn’t explain to me what the needles were before they gave them… They just kind of brought me to the appointments and gave the kids needles. Not even my consent or anything… I am like, “What if there is a certain kind that I don’t want them to have, or they don’t necessarily need?” … I know they would have given [options] to older parents.

Another mother shared: “I am assuming they thought that because I was nineteen, I didn’t know what I was doing. So the typical, people thinking that young moms are bad moms. Which is silly.” Both mothers attributed this discriminatory care to their young age, and believed older mothers received more respectful and informed care.

##### Approaches to care

Equally important to building relationships was how a PCP approached their provision of primary care. Mothers felt more confident caring for the health of their infants when PCPs provided anticipatory guidance and collaborative care. In the same way, mothers generally felt that PCPs could improve how they approach the health of Indigenous mothers and infants by providing culturally relevant care.

First, PCPs who provided anticipatory guidance demonstrated their ability to predict which knowledge and skills mothers needed to care for their infant (Hsu, Lee, Lai, Tsai, & Chiu, 2018). Mothers whose PCPs counselled them on what to expect also said they were more confident in their understanding of their infant’s health, needs and treatment plans. Those who had seemingly not been provided anticipatory guidance felt insecure in their knowledge of their infant’s health, and worried about what came next in terms of monitoring and treating health issues. One mother explained that she would rather her infant’s PCP share “…this is what is happening, this is what we are going to do, this is what is going to happen at the next appointment”. Health providers recognized the need for anticipatory guidance. They also explained that this type of care was time-consuming, requiring them to book longer appointments with infants and mothers to ensure sufficient time to spend with families.

Second, mothers expressed the importance of an engaging PCP who took a collaborative approach. Collaboration meant allowing mothers to hold their infants during uncomfortable and/or painful exams and procedures, as well as involving them in decisions about their infant’s health. Mothers felt their values and beliefs were supported when they engaged in decision-making with their infant’s PCP.

Third, mothers strongly believed that their infant’s PCPs should provide culturally relevant care. They felt this could be accomplished by having an understanding and appreciation of Indigenous history, and by valuing and supporting cultural and spiritual beliefs as they relate to health and well-being. Mothers recognized the inability of most PCPs to incorporate traditional Indigenous medicine into their treatment plans but expected PCPs to respect its place in health and healing, and to provide links to cultural resources in the city. An excerpt from the discussion group describes how mothers believed an understanding of how culture and associated trauma from colonization influences negative stereotypes of Indigenous people:

Participant mother 1 It bothers me just overall how uneducated the health care system…Like I said, we are the original people, you should know so much about our history and it should be so incorporated.

Participant mother 2 …I still meet people every day that know nothing about it. I am like really? There is a reason for that drunken person on the bench.

Participant mother 1 I know. And it’s true then they look at them and think ugh, drunk Indian.

Participant mother 2 Or they think they’re lazy.

Participant mother 3 If you have to legally take your WHMIS [workplace health and safety program] to work then why shouldn’t you be able to take something that makes you culturally sensitive?

Primary care providers shared conflicting stories of their ability to provide culturally relevant care. Some providers had taken cultural training in their workplace, while others recognized their need for cultural training to understand of the impacts of colonization and better familiarize themselves of the city’s culturally based resources for Indigenous people. The provision of culturally relevant care is important to mothers because they encounter negative stereotypes about Indigenous people in their interactions with their infant’s PCPs. This subsequently leads to negative encounters and affects their decisions to return for care.

## Discussion

**Figure 1.**
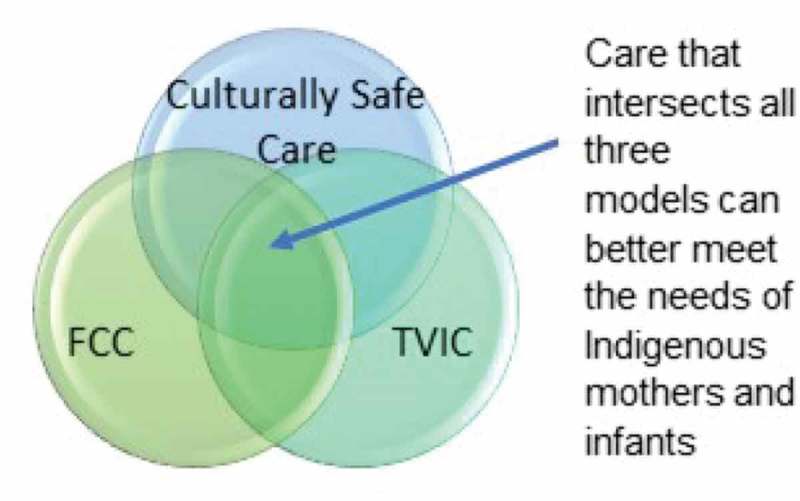
The intersection of culturally safe, TVIC and FCC models of care.

This is the first qualitative study to ask Indigenous mothers living in a Canadian urban centre how they select and use primary health services to care for the health needs of their infants. This understanding is vital to informing how PCPs provide care for Indigenous mothers and infants in Hamilton, and how access and use of primary care services might be improved. Results speak to the challenges experienced by mothers, which we believe may be mitigated by taking an approach to primary care that intersects culturally safe, trauma and violence-informed (TVIC), and family-centred care (FCC) models (Figure 1).

While the results describe barriers to care experienced by other marginalized groups, it is important to recognize that the contextual factors influencing the lives of mothers and infants in this study are largely a result of racist and discriminatory policies that stem from colonization. The Indian Act of 1876 disproportionately affected Indigenous women; until 1985 those who married non-Indigenous men would lose their Indian status and its associated benefits (including living in their reserve communities), and women were not allowed to participate in band governance (University of British Columbia, 2009). This has led to more women than men living off-reserve in urban areas where they more commonly lead single-parent families, experience economic disadvantage and have fewer educational opportunities (Browne et al., 2016; C. Reading, 2015). Many of the mothers in this study lived in disadvantaged neighbourhoods. These very neighbourhoods are an example of structural violence; people experiencing poverty have a higher likelihood of exposure to pollutants, noise, and inadequate housing in disadvantaged neighbourhoods, which negatively impact child development and overall health and well-being (Conroy, Sandel, & Zuckerman, 2010; Hertzman, 2010). Therefore, the financial penalties imposed on mothers by primary caregivers for taking their infants to a walk-in clinic or missing an appointment represent examples of how living within a context of poverty negatively impacts their access to health care, and how health providers perpetuate harm by discriminatory policies and practices. Mothers described the need for PCPs to understand how contextual factors, including colonization, the residential school legacy and other historical events uniquely impact Indigenous people, including health behaviour, use of health services and health outcomes.

The literature suggests that health providers can accomplish these goals through taking a culturally safe approach to care (Browne et al., 2016; The Truth and Reconciliation Commission of Canada, 2015). Culturally safe care was initially described by Maori nurses in New Zealand who believed it imperative that health professionals understood not only the differences between their own beliefs and values and those of their patients, but also recognized the unintended potential to cause harm to patients as a result of their ignorance (Papps & Ramsden, 1996). The urgent need for culturally safe health care practices has been recognized in the *Calls to Action* by the Truth and Reconciliation Commission of Canada (TRC), which instructs health professionals to become educated on the history of colonization in Canada and the continuing impact of historical trauma on the health of Indigenous people today (The Truth and Reconciliation Commission of Canada, 2015). With an accurate understanding and appreciation of the history of Indigenous people in Canada, health professionals are empowered to abolish negative stereotypes, promote equitable and safe access to health care, most certainly benefiting the health outcomes of patients. Similarly, PCPs who have an appreciation of Indigenous history and culture can support mothers in meeting their infant’s cultural needs by respecting the role of traditional ceremonies and medicines in health and wellness. Supporting the cultural needs of children, especially in urban areas, has been demonstrated as important to their development of self-esteem and confidence (Gerlach, Browne, & Suto, 2016; Priest, Mackean, Davis, Briggs, & Waters, 2012). Primary care providers in this study, however, were largely unaware of cultural resources in the community, which demonstrates the need for information sharing and collaboration with Indigenous organizations in the community.

Applying TVIC principals to care is another way that PCPs can make important contributions to the health and well-being of Indigenous families. Primary care providers who practice TVIC are aware of how organizational policies and how they provide care have the potential to cause further harm and trauma to patients. To lessen this risk, they are mindful of how their affects others and advocate for organizational policies that promote safe care.

The mothers in this study articulated three important areas in which TVIC should be applied to reduce the risk of perpetuating harm and trauma during health care encounters. First, young mothers shared how experiences of discrimination relating to their age negatively impacted their access to health care for their infants. This has been demonstrated in other research as well (Ballantyne, Benzies, Rosenbaum, & Lodha, 2015; Martens et al., 2012). Indigenous mothers tend to be younger than non-Indigenous mothers; more than a quarter of First Nations children (ages 5 and under) living off-reserve having mothers ages 15 to 24 years compared to just 8% of non-Indigenous children (Smylie & Adomako, 2009). It is also known that teenage pregnancy can be detrimental to a woman’s health, as it increases her vulnerability to poverty, single parenthood and depression, and lowers her chances of obtaining a high school diploma, and subsequent employment (National Collaborating Centre for Aboriginal Health, 2012). As such, PCPs providing TVIC can lessen the potential for trauma and harm by providing safe places for intimate exams and sensitive conversations while taking care to not make assumptions about the parenting abilities of young mothers (Varcoe, Wathen, Ford-Gilboe, Smye, & Browne, 2016).

Second, the involvement of child protection services is especially traumatic for Indigenous families with the history of the residential school legacy and the Sixties Scoop in which children were forcibly removed from families, leading to the over-representation of Indigenous children in the care of child protection services (The Truth and Reconciliation Commission of Canada, 2015). As described by mothers in this study, threatening the involvement of child protection services as a punitive action for missed appointments can be extremely harmful as it may elicit feelings of fear and trauma, and lead to mothers not returning for care; potentially putting their own health and the health of their infants at risk (Denison, Varcoe, & Browne, 2013). Indeed, the mothers in this study shared their feelings of fear, shame, anger and mistrust of their infant’s PCPs when these instances occurred.

Third, racist policies in health care represent structural violence that result in traumatic experiences for Indigenous mothers and infants. This trauma can be intergenerational; passing down through generations and negatively impacting the mental and physical health of children (Varcoe et al., 2016). Not every mother in this study described experiences of racism in health care, some may have been cared for by contextually aware health providers or have otherwise been protected by policies that make identifying Indigenous people living off-reserve difficult. People are not obligated to disclose their Indigenous identity in Canada, and many choose not to for fear of receiving racialized treatment. Racist policies continue to be evident in health care today, including which Indigenous people are eligible for extended health benefits and which are not, and abolishing these policies requires significant changes to the Indian Act which defines who is granted Indigenous status in discriminatory ways. Eradicating these policies requires changes to political and social agendas which will take time, and ultimately requires that all Canadians are educated on the history of Indigenous people and colonization in Canada to significantly reduce racism and discrimination. In the meantime, everyone can do their part to eliminate racist and discriminatory practices by becoming allies, fighting for equity and the acknowledgement of Indigenous rights.

Applying TVIC principles to the care of Indigenous mothers and infants, PCPs can take a strengths-based approach, build trusting relationships and involve social and community supports that can help to counter the presence of risk factors (Varcoe et al., 2016). Providing anticipatory guidance can effectively promote positive health interactions, building trusting relationships, reducing distress and enabling mothers to make informed choices to promote their child’s health and development (Hsu et al., 2018). Mothers who are well-informed and know what to expect for the care of their infant (ie. Follow-up visits, developmental milestones, safety precautions) are more confident parents (Hsu et al., 2018). Clinics can promote confidentiality, and ensure safe and private areas are available for sensitive conversations (Varcoe et al., 2016). Organizations can provide a welcoming space, with receptionists who are also trained in culturally safe and TVIC, as this sets the tone of the clinic and influences patient experience (Varcoe et al., 2016).

Finally, a FCC approach to primary care is an important way to engage the family unit and improve access to care. Initially described in the 1950s, FCC advocates for the bringing together of families through the recognition of a child’s family as the primary caregivers and constant within a child’s life (Harrison, 2010). A FCC approach should influence all levels of health care from health policy to practice, integrating the family into decision-making and care provision for their child, and recognizing the diversity of families and their need for holistic care (Harrison, 2010). While most health providers believe FCC is inherently good for children and families, they remain unclear on how best to implement FCC principles, particularly those related to culturally safe care (Dennis, Baxter, Ploeg, & Blatz, 2017; Shields, 2015). The results of this study contribute to this gap in understanding and provide specific strategies to addressing culturally safe care with Indigenous families.

Perhaps most importantly, and as advocated for by mothers, FCC requires a relationship that is built on trust, enabled through continuity of care, and maintained without judgment, racism or discrimination. In an integrative review of trust between a patient and PCP, Murray and McCrone (2015) suggested its presence can increase a patient’s participation in their care, their use of primary care services and their satisfaction with these services. Mothers described several ways that PCPs can build trusting relationships, also demonstrated in the literature: effective communication skills—that is, listening, understanding patient experiences and context—as well as displaying empathy and spending the necessary time with patients to adequately address their concerns (Murray & McCrone, 2015). A FCC perspective emphasizes a collaborative approach to the care of infants, an additional avenue to the establishment of trusting relationships. This enables a PCP to work in a family’s best interest and knowing infants are truly cared for, helps mothers impacted by social inequity and structural violence to feel safe in the healthcare environment. Ensuring infants are seen by the same PCP at each healthcare encounter also contributes to building trusting relationships, and has been found to reduce emergency hospital admissions (Huntley et al., 2014; Murray & McCrone, 2015). A friendly and welcoming environment that minimizes power imbalances between PCP and mothers is important to an FCC approach to care, building trust and promoting equitable access to care (Murray & McCrone, 2015; Varcoe et al., 2016).

Primary care that integrates culturally safe, TVIC and FCC models is an effective way of improving access to care and promoting positive interactions between PCPs and Indigenous mothers and infants. A culturally safe lens provides PCPs with an understanding of how culture impacts health and well-being, while a TVIC lens can help health providers appreciate the implications of structural violence with its resulting trauma and inequity. While a TVIC approach includes culturally safe care principles, primary care for Indigenous people requires that PCPs have a thorough understanding of the role of culture in health and well-being as well as the importance of holistic care. Used in combination, these three approaches can help to balance social inequities, improve access to primary care and improve health outcomes for Indigenous infants and families.

## Limitations

There are several limitations to this study. First, this study included Indigenous mothers living in an urban area off-reserve, and not all identified strongly with their culture. Their experiences, therefore, may not reflect those of Indigenous mothers who are disconnected or seeking to reconnect with their culture. Next, the perspectives of fathers and parents of other family models were not elicited in this study, yet they offer important perspectives to the provision of primary care for Indigenous infants. Finally, the health providers who took part in this study likely participated because they believed they provided acceptable or exemplary care. Their experiences, therefore, may not reflect those of all health providers caring for Indigenous patients, and may not reflect the experiences of all Indigenous mothers seeking care for their infants, and mostly likely represent the best case.

## Conclusions

This study is the first qualitative study in an urban centre in Canada to ask Indigenous mothers how they select and use primary care to meet the health needs of their infants. The experiences of Indigenous mothers reflect the importance of intersecting three models of care; culturally safe care, TVIC and FCC. Through the TRC and its mandated Calls to Action, health providers are encouraged to engage in education to enhance their awareness of how colonization has resulted in social inequities, structural violence and trauma for Indigenous people. This research resulted in a remarkable, though perhaps not surprising, finding. The suggestions made by Indigenous mothers on how best to improve interactions with health providers—and consequently also improve health care access for their infants and families—are generally within the power of the individual. That is to say, while critical changes are necessary at policy and organizational levels, PCPs can improve access to care for Indigenous mothers and infants in important ways as well, by implementing these insights to the way they provide care. These changes offer the potential of a meaningful and positive impact on the health and well-being of Indigenous infants during their early development, a period when access to primary care is critical to health outcomes over the life span.

## Notes

1. Early childhood development services consist of parenting supports that promote parent-infant attachment and healthy infant growth and development.
